# Thermodynamic Work of High-Grade Uterine Prolapse Patients Undergoing Transvaginal Mesh Repair with Total Hysterectomy

**DOI:** 10.3390/bioengineering11090875

**Published:** 2024-08-28

**Authors:** Hui-Hsuan Lau, Cheng-Yuan Lai, Ming-Chun Hsieh, Hsien-Yu Peng, Dylan Chou, Tsung-Hsien Su, Jie-Jen Lee, Tzer-Bin Lin

**Affiliations:** 1 Division of Urogynecology, Department of Obstetrics and Gynecology, Mackay Memorial Hospital, Taipei 104217, Taiwan; huihsuan1220@gmail.com (H.-H.L.); drthsu571@gmail.com (T.-H.S.); 2 Department of Medicine, Mackay Medical College, New Taipei 25245, Taiwan; ming.chun2@gmail.com (M.-C.H.); dylanchou@mmc.edu.tw (D.C.); jjlee918@mmc.edu.tw (J.-J.L.); 3 Institute of Biomedical Sciences, Mackay Medical College, New Taipei 25245, Taiwan; cheng.yuan5@gmail.com; 4 Department of Surgery, Mackay Memorial Hospital, Taipei 104217, Taiwan; 5 Institute of Translational Medicine and New Drug Development, College of Medicine, China Medical University, Taichung 404328, Taiwan; 6 Department of Physiology, School of Medicine, College of Medicine, Taipei Medical University, Taipei 110301, Taiwan; 7 Graduate Institute of Biomedical Electronics and Bioinformatics, National Taiwan University, Taipei 10617, Taiwan

**Keywords:** thermodynamics, workload, resistance, pelvic organ prolapse, pelvic floor repair

## Abstract

The objective benefit of transvaginal mesh with concomitant total hysterectomy (TVM-HTX) repair to high-grade uterine prolapse (UP) patients has not been fully established. This study aimed to clarify the impact of TVM-HTX on the voiding function of high-grade UP patients by comparing pre- and post-operatively measured pressure–flow and pressure–volume investigations. Urodynamic and thermodynamic studies were conducted on 15 high-grade UP patients (stage III/IV, POP-Q system) who underwent TVM-HTX (January 2019–December 2022) in a tertiary-care university hospital. The parameters analyzed included voiding resistance (Rvod), voiding pressure (Pvod), voiding flow (Fvod), voided volume (Vvod), voiding time (Tvod), and the trajectory-enclosed area of the pressure–volume loop (Apv). Post-operative results showed a significant reduction in Rvod (*p* < 0.001, N = 15), Pvod (*p* = 0.021, N = 15), and Apv (*p* = 0.006, N = 15), along with an increase in Fvod (*p* = 0.003, N = 15) and a decrease in Tvod (*p* < 0.001, N = 15). The operation-associated changes in Rvod (ΔRvod) correlated with alterations in Pvod and Fvod (ΔPvod and ΔFvod, r = 0.444, *p* = 0.004, r = 0.717, *p* = 0.003, respectively; both N = 15); ΔFvod correlated with the change in Tvod (ΔTvod, r = 0.629, *p* = 0.012, N = 15) but not with that in ΔVvod (r = 0.166, *p* = 0.555, N = 15). Changes in Apv (ΔApv) correlated with ΔPvod (r = 0.563, *p* = 0.029, N = 15) but not with ΔVvod (r = 0.353, *p* = 0.197, N = 15). Collectively, TVM-HTX reduced the voiding resistance, which improved the voiding efficacy and decreased the pressure gradient required for driving urine flow, thereby lessening the bladder’s workload.

## 1. Introduction

Pelvic organ prolapse (POP), characterized by the descent of pelvic structures into the vagina due to weakened ligament or muscle, significantly impacts the quality of life and well-being of menopausal-age women [1] because POP commonly affects women aged higher than 70 years old [2,3] and displays a higher incidence in menopausal compared to pre-menopausal females [4]. Among the subcategories of POP, uterine prolapse (UP), where the uterus, cervix, or vaginal apex descends towards the vaginal opening, predominantly affects menopausal women with a history of vaginal deliveries [5]. Although the uterus is considered to play a passive role in UP development [6] and the benefit of a hysterectomy in correcting apical support defects remains debated [7], the surgical reconstruction of UP typically involves a hysterectomy combined with vaginal vault suspension [8], and there are approximately 74,000 cases of hysterectomy for UP repair performed annually in the United States [9].

To provide sufficient apical support preventing prolapse of the vaginal stump [10], native tissue was first used for vaginal vault suspension after a hysterectomy [11]. Nevertheless, because the pelvic structure progressively becomes weaker, bringing about a high recurrence rate following age increases [12], synthetic mesh repair is advocated for high-grade prolapse patients in order to provide adequate support [13] because it displays lower rates of recurrent prolapse compared with native tissue repair [14]. Although the U.S. Food and Drug Administration (US FDA) is concerned about the long-term safety of mesh repair [15,16], some mesh kits [17,18,19] are viable options for POP surgery in Asia and continental Europe, because they offer a successful anatomical reconstruction and satisfied objective outcomes [20]. Nevertheless, while our publication demonstrates that transvaginal mesh repair improves the voiding function of POP patients, whether transvaginal mesh with concomitant hysterectomy (TVM-HTX) objectively benefits the voiding function of high-grade UP patients [21] has not been investigated.

The bladder outlet obstruction [22] caused by the kinking or compression of the urethra [23] is presumed to underlie the voiding difficulties in POP patients [24]. Considering bladder outlet obstruction manifests as an increased voiding resistance during emission [25], we queried if TVM-HTX benefits the voiding function of high-grade UP patients by diminishing the voiding resistance. For this purpose, we retrospectively reviewed the urodynamic data of UP patients measured pre- and post-operatively. The impact of TVM-HTX on voiding resistance and associated parameters was analyzed. Moreover, whether the TVM-HTX-modified resistance is associated with altered voiding work in high-grade UP patients was investigated using a pressure–volume analysis (PVA) of voiding because our animal [26,27] and clinical [28,29] studies have demonstrated the loop of the trajectory in the PVA graphically and conceptually assessing the thermodynamic work of voiding cycles.

## 2. Materials and Methods

### 2.1. Study Design

This study was reviewed, approved by the ethics committee of Mackay Memorial Hospital, Taipei, Taiwan (22MMHIS361e; 8 December 2022), and registered in ClinicalTrials.gov (NCT05682989). We reviewed urodynamic data from patients who underwent a transvaginal total hysterectomy with mesh repair for symptomatic stage III or IV UP (POP-Q system) from January 2019 to December 2022 in Mackay Memorial Hospital. Patients with a history of fistula, pelvic radiation, and concurrent anti-incontinence surgery, and failed to complete pre- and post-operative follow-ups were excluded from the analysis.

### 2.2. Surgical Procedures

The hysterectomy was performed with the patient in the dorsal lithotomy position. Briefly, a circumferential incision was made around the cervix; and ligaments and attachments were separated to free the uterus, which was then removed [30]. Adapted from previous publications [19,31], transvaginal mesh repair was carried out using a surgical kit (Surelift^®^, Neomedic International, Barcelona, Spain). Concisely, the vesico-vaginal space was dissected and identified. Two central arms of the mesh were cut off as modification, two posterior arms were fixed to the sacrospinous ligaments, and the other two anterior arms were penetrated through the obturator foramens in an outside-in fashion. After the mesh was adjusted in a tension-free manner, sutures were made beneath the bladder neck and near the vaginal vault. After mesh implantation has completed, the vaginal cuff was closed with a suture.

### 2.3. Pressure–Flow/–Volume Analyses

Urodynamic studies in which warm saline (37 °C) was infused into the bladder of patients (80 mL/min) were recorded (MMS UD-200, Medical Measurement System, Enschede, The Netherlands) and analyzed (Biopac MP36, Biopac Systems, Santa Barbra, CA, USA) using computer systems. Parameters recorded included vesical pressure (Pves; cmH_2_O), abdominal pressure (Pabd; cmH_2_O), detrusor pressure (Pdet=Pves-Pabd; cmH_2_O), urethral flow (Flow; mL/s), infused volume (Vinf; mL), voided volume (Vvod; mL), and intra-vesical volume (Vive = Vinf − Vvod; mL); these were recorded online. Mean voiding pressure (Pvod, the mean Pdet during fluid emission; cmH_2_O), voiding time (Tvod, the duration of fluid emission; sec), mean voiding flow (Fvod = Vvod/Tvod;), and mean voiding resistance (Rvod = Pvod/(Vvod/Tvod); cmH_2_O-s/mL) were analyzed offline. The pressure–volume analysis (PVA) of voiding cycles was established by plotting Pdet against Vive [26,27,28,29]. The trajectory-enclosed area (Apv; cmH_2_O-mL) was analyzed using an image processing program (Image J 1.5.3, LOCI, Madison, WI, USA).

### 2.4. Statistical Analysis

Patient demographics were summarized using descriptive statistics. Data were expressed as mean ± SEM, and differences between groups was assessed using paired Student’s *t*-tests, with significance set at *p* < 0.05.

## 3. Results

### 3.1. Baseline Database of Patients

Cystometry evaluations of 15 high-grade UP patients (mean age = 64.62 ± 1.68 years old), who pre-operatively displayed stage III or IV prolapse (POP-Q system), were reviewed and analyzed. As shown in Table 1, urodynamic evaluations were carried out at a mean of 32.86 ± 9.64 days before and 135.20 ± 19.06 days after the operation.

### 3.2. TVM-HTX Diminishes Voiding Resistance

**Figure 1:**
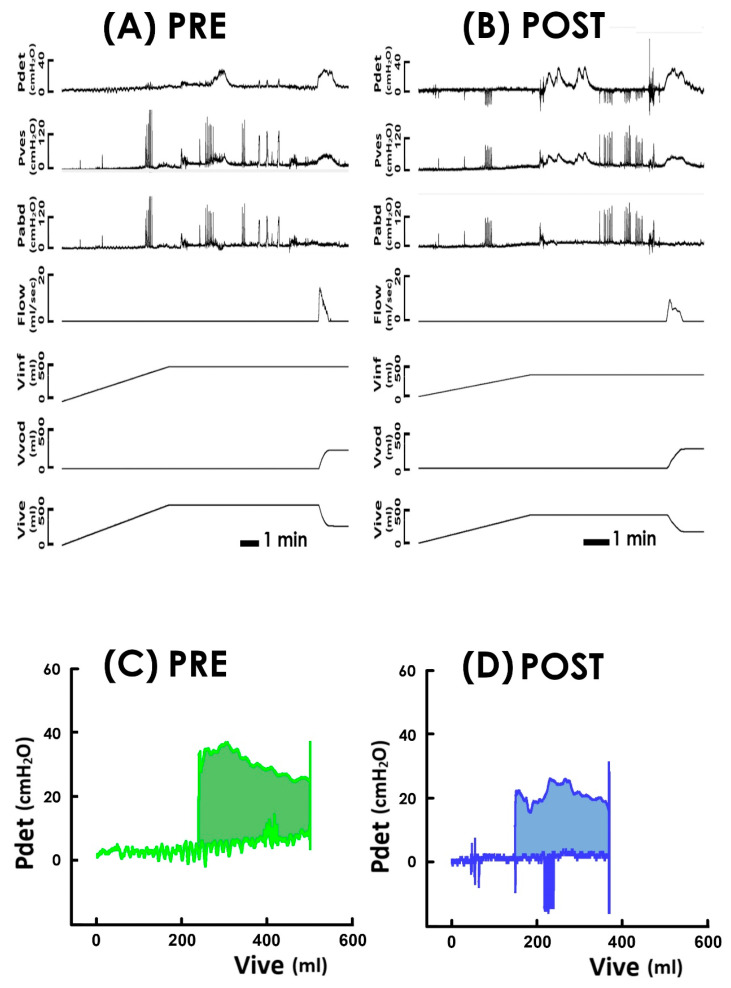
**Pressure–flow/–volume analyses of voiding in response to TVM-HTX.** (**A**) **PRE and** (**B**) **POST** Representative cystometry of a high-grade UP patient measured pre- and post-operatively. Pdet: detrusor pressure, Pves: vesical pressure, Pabd: abdominal pressure, Flow: urethral flow, Vinf: infused volume, Vvod: voided volume, and Vive: intra-vesical volume. (**C**) **PRE** and (**D**) **POST** pre- (green) and post-operatively (blue) PVA derived from the cystometry. Hatched areas denote the trajectory-enclosed areas.

**Figure 2:**
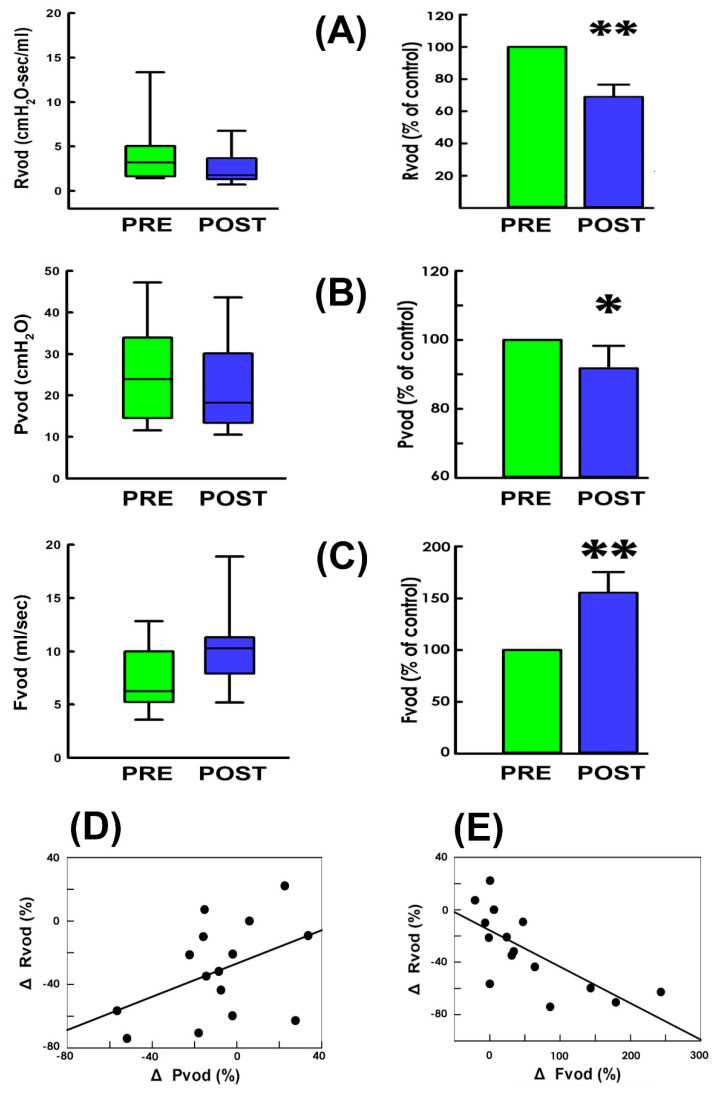
**Voiding resistance, pressure, and flow in response to TVM-HTX.** Box plot (left) and mean values (right) of (**A**) voiding resistance (Rvod), (**B**) voiding pressure (Pvod), and (**C**) voiding flow (Fvod) measured pre- and post-operatively. (PRE: green and POST: blue. ** *p* < 0.001, * *p* = 0.021, ** *p* = 0.003, respectively, vs. PRE, all N = 15. (**D**,**E**) Correlation analyses of the operation-associated change in voiding resistance (ΔRvod) with the changes in voiding pressure (ΔPvod; r = 0.444, *p* = 0.004, N = 15) and voiding flow (ΔFvod; r = 0.717, *p* = 0.003, N = 15), respectively.

Because enhanced urethral resistance caused by outlet kinking or compression underlies the voiding deficit in UP patients [23], we first examined if TVM-HTX diminishes voiding resistance in high-grade UP patients. Derived from the representative cystometry (Figure 1A,B) and PVAs (Figure 1C,D) assayed pre- and post-operatively (PRE and POST, respectively), the summarized data demonstrated TVM-HTX consistently decreased the mean voiding resistance (Figure 2A,C Rvod) in most patients (13 out of 15 (13/15)) and significantly decreased the mean Rvod of the patient (*p* < 0.001 vs. PRE, N = 15) when compared with the pre-operative control, indicating TVM-HTX post-operatively diminished the voiding resistance of UP patients.

### 3.3. TVM-HTX Modifies Voiding Pressure and Flow

Because the voiding resistance is computed by dividing the voiding pressure by the flow rate, we investigated if the TVM-HTX-diminished resistance is accompanied by a modified voiding pressure or flow rate. The summarized data demonstrated that, when compared with the pre-operative control (Figure 2B PRE), TVM-HTX consistently decreased the mean voiding pressure (Pvod) in 11 out of 15 patients and significantly decreased the mean Pvod of the patient (*p* = 0.021 vs. PRE, N = 15), indicating the bladder post-operatively developed a lower pressure gradient required for driving urine flow. Moreover, TVM-HTX consistently increased the mean flow rate of voiding in most patients (Figure 2C Fvod; 12/15) and significantly increased the mean Fvod of the patient compared with the pre-operative control (*p* = 0.003 vs. PRE, N = 15), indicating TVM-HTX post-operatively prompted urine emission by bringing about an elevated flow rate.

To specify the relationships between the TVM-HTX-associated Rvod change (ΔRvod) and the Pvod and Fvod changes (ΔPvod and ΔPvod, respectively), we assayed the correlation of ΔRvod with ΔPvod and ΔPvod. Bivariate analyses demonstrated the ΔRvod correlated with the ΔPvod (Figure 2D) and the ΔFvod (Figure 2E). These findings revealed the TVM-HTX-diminished voiding resistance was associated with decreased voiding pressure and increased voiding flow.

### 3.4. TVM-HTX Shortens Voiding Time

**Figure 3:**
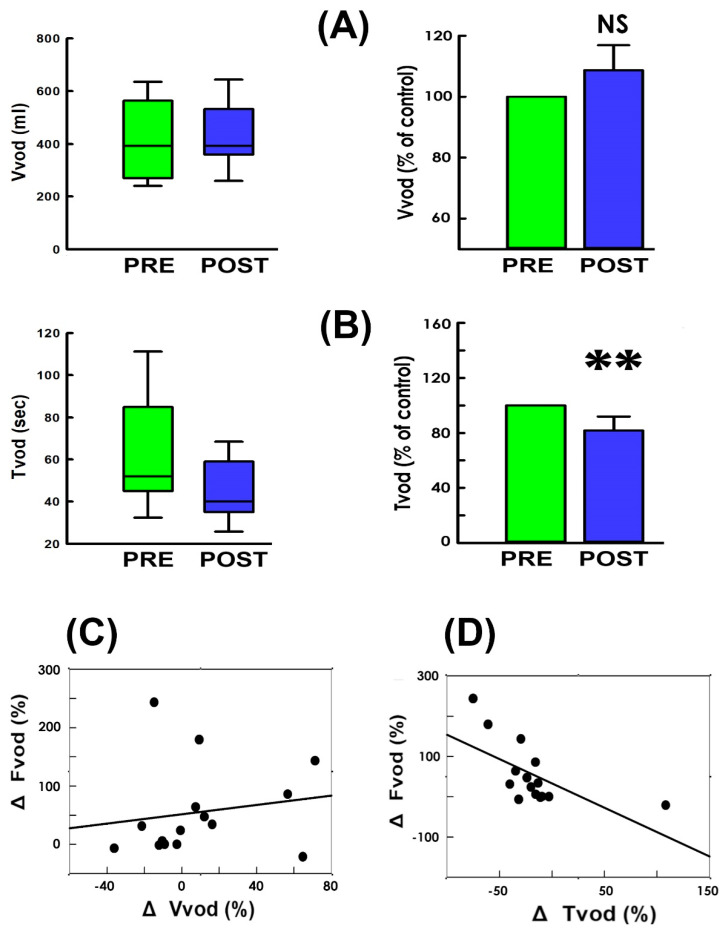
**Voided volume and voiding time in response to TVM-HTX.** Box plot (left) and mean values (right) of (**A**) voided volume (Vvod) and (**B**) voiding time (Tvod) measured pre- and post-operatively (PRE: green and POST: blue; NS *p* = 0.756, ** *p* < 0.001, respectively, vs. PRE, both N = 15). (**C**,**D**) Correlation analyses of the operation-associated change in voiding flow (ΔFvod) with the changes in voided volume (ΔVvod; r = 0.166, *p* = 0.555, N = 15) and voiding time (ΔTvod; r = 0.629, *p* = 0.012, N = 15), respectively.

Because the flow rate is calculated by dividing the voided volume (Vvod) by the voiding time (Tvod), we next assayed the impact of TVM-HTX on the Vvod and Tvod. The summarized data demonstrated that, when compared with the pre-operative control (Figure 3A PRE), TVM-HTX neither resulted in a consistent Vvod change in patients nor significantly affected the mean Vvod of the patient (*p* = 0.756 vs. PRE, N = 15). In contrast, TVM-HTX post-operatively decreased Tvod in most patients (Figure 3B. 14/15) and significantly decreased the mean Tvod of the patient (*p* < 0.001 vs. PRE, N = 15) when compared with the pre-operative control.

Moreover, bivariate analyses demonstrated the TVM-HTX-associated Fvod change (ΔFvod) failed to correlate with the Vvod change (ΔVvod; Figure 3C); but it correlated with the ΔTvod (Figure 3D). These findings reveal that the TVM-HTX-elevated voiding flow was accompanied by a shortened voiding time and a negligibly affected voided volume.

### 3.5. TVM-HTX Lessens Voiding Workload

**Figure 4:**
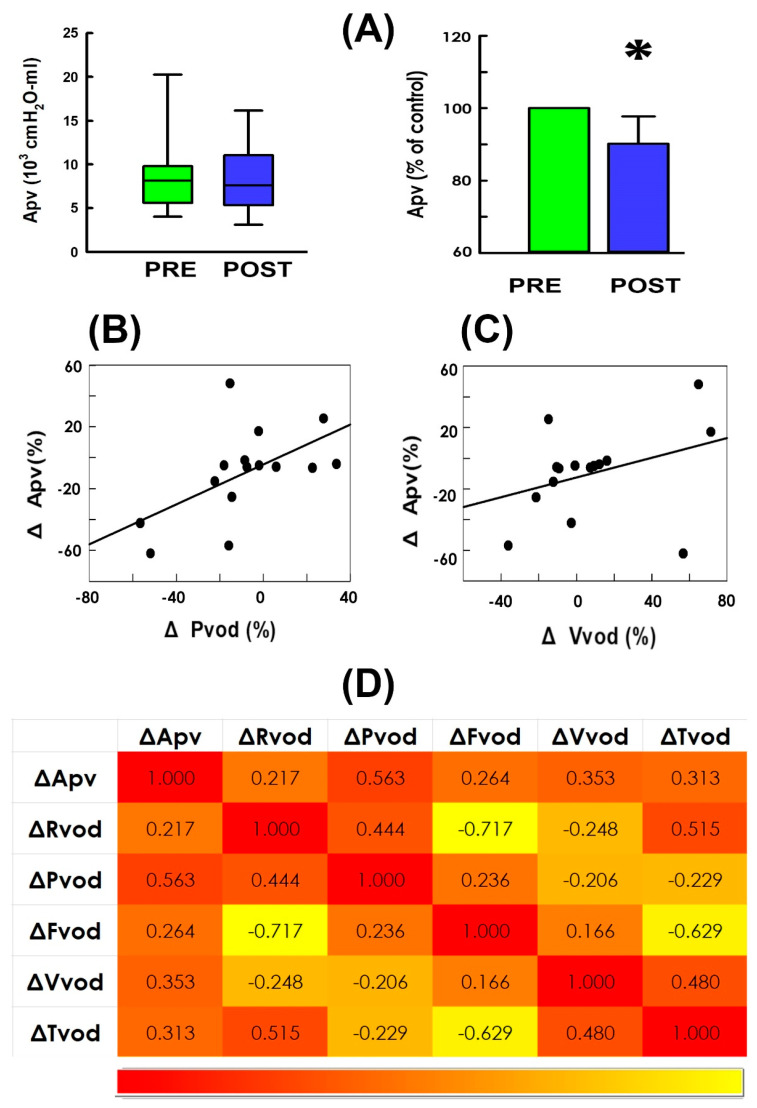
**Trajectory-enclosed area in response to TVM-HTX.** (**A**) Box plot (left) and mean values (right) of the loop-enclosed area (Apv) measured pre- and post-operatively (PRE: green and POST: blue. * *p* = 0.006, vs. PRE, N = 15). (**B**,**C**) Correlation analyses of the operation-associated change in Apv (ΔApv) with the change in voiding pressure (ΔPvod; r = 0.563, *p* = 0.029, N = 15) and voided volume (ΔVvod; r = 0.353, *p* = 0.197, N = 15), respectively. (**D**) Heatmap displaying Pearson’s correlation analysis r values between all pairs of variables. Red and yellow colors represent positive and negative r, respectively.

Having observed that TVM-HTX effectively diminished the voiding resistance, and the bladder post-operatively developed a decreased pressure, while it drove urine with a higher emission rate, we wonder if these findings collectively reveal that TVM-HTX lessens the voiding workload of UP patients. For this purpose, we assayed the trajectory-enclosed area (Apv; Figure 1C,D hatched areas) in pressure–volume analyses (PVAs) which is presumed to represent the work of the voiding cycle in animal [26] and clinical [28,29] studies. When compared with the pre-operative control (Figure 2C PRE), the illustrative PVA demonstrated that TVM-HTX markedly depressed the top border and shifted the loop slightly to the left while minimally affecting the volume difference between the left and the right borders (Figure 2D POST), confirming that the cystometry demonstrated a decreased Pvod with a barely affected Vvod after operation. Moreover, as the Apv is the integral of the pressure with respect to volume, a decreased Pvod with an unaffected Vvod ultimately resulted in a decreased Apv. The operation-associated Apv decrement was confirmed by the summarized data demonstrating that, when compared to the pre-operative control (Figure 4A PRE), TVM-HTX post-operatively decreased Apv in most patients (12/15) and significantly decreased the mean Apv of the patient (*p* = 0.006 vs. PRE, N = 15).

Considering Apv is the integral of Pdet with Vive, and the above results demonstrated TVM-HTX post-operatively decreased Pvod without markedly affecting Vvod, we analyze the relationship between the operation-associated Apv change (ΔApv) and the Pvod change (ΔPvod) and Vvod change (ΔVvod) to elucidate if TVM-HTX lessened the voiding work via diminishing the developed pressure. As anticipated, ΔApv was shown to be moderately correlated with ΔPvod (Figure 4B) rather than ΔVvod (Figure 4C), indicating the TVM-HTX-decreased Apv was mainly attributed to the diminished Pvod. Moreover, the correlation coefficients between all pairs of parameters are summarized as a heatmap (Figure 4D)

## 4. Discussion

### 4.1. TVM-HTX for UP Patients

When the symptoms of UP become debilitating and non-invasive options and surgical repair are not appropriate, a hysterectomy to remove the prolapsed uterus may be recommended, even in the absence of uterine disease [8]. While there are exceptions—such as the patient’s desire for future fertility, concerns about sexual function, or the surgical risks associated with a hysterectomy [32,33]—the procedure is generally considered favorable for UP reconstruction, due to its low intra-operative risk and potential to reduce the morbidity and mortality associated with endometrial and cervical cancer [8]. However, it is essential that we objectively evaluate the therapeutic effect of a concomitant hysterectomy during prolapse surgery to provide clinicians with comprehensive information for surgical decision-making.

A hysterectomy can be performed intra-vaginally, laparoscopically, or robotically through an abdominal incision, or laparotomically [8]. Among these, the intra-vaginal approach is often preferred because it avoids an abdominal incision and scar, allows access to the peritoneal cavity for high vaginal vault suspensions, and reduces the risk of cervical elongation, which can cause recurrent prolapse or dyspareunia [7]. Accompanied by a concurrent hysterectomy, the surgical repair of UP most commonly involves vaginal vault suspension [8]. Despite FDA warnings about the long-term safety of mesh repair in prolapse surgery [15,16], mesh repair remains a viable option in Asia and continental Europe due to its reportedly favorable anatomical reconstruction and low complication rates [34].

### 4.2. TVM-HTX Diminishes Voiding Resistance

Consistent with studies demonstrating that transvaginal mesh implantation offers successful anatomical restoration and relief of subjective symptoms [20], and our previous publication recently showed that transvaginal mesh repair objectively improves the voiding function of POP patients [28], resulting in the current study indicating that TVM-HTX benefits the voiding function of high-grade UP patients by consistently and significantly diminishing the voiding resistance. Given that voiding difficulties in POP patients are often attributed to aberrantly enhanced urethral resistance [24] caused by the kinking and/or compression coming from the prolapsed structure [23], our results suggest that TVM-HTX restores the anatomical confines of organs neighboring the urethra, therefore relieving the outlet kinking and/or compression.

Moreover, because the voiding resistance was computed by dividing the voiding pressure by the voiding flow, we further characterized the impact of TVM-HTX on the voiding pressure and volume. The results of cystometry demonstrated that the diminished voiding resistance following TVM-HTX was accompanied by a decreased voiding pressure, a result confirmed by the PVA analysis showing that the top border of the loop, representing the voiding pressure, was markedly depressed post-operatively. Moreover, the diminished voiding resistance was associated with consistent and significant increments in the flow rate during voiding. As bivariate analyses demonstrated that the change in voiding resistance correlated with both the changes in voiding pressure and voiding flow, these findings collectively reveal that the operation-diminished voiding resistance brings about a reduced voiding pressure accompanied by an enhanced flow rate during voiding. We suggest that the diminished voiding resistance following TVM-HTX reduced the pressure gradient driving urine flow to lessen the voiding workload, and prompted urine emission to increase voiding efficacy.

### 4.3. Lessened Voiding Workload

Our proposal is supported by the following: first, the data from cystometry demonstrated that TVM-HTX consistently and significantly decreased the voiding pressure but negligibly affected the voided volume, a finding confirmed by the PVA, in which the top border was markedly reduced with a trivially affected intercept between the left and right borders, representing the voided volume. Because Apv is defined as the integral of the pressure with respect to volume in a voiding cycle, these results reveal that the operation-decreased Apv was primarily driven by a decreased voiding pressure. This suggestion is confirmed by the correlation analysis showing that the operation-induced Apv decrement correlated with the decrement in voiding pressure. Further considering the Apv is presumed to represent the thermodynamic work performed in a voiding cycle [26,27,28,29], these findings collectively support our proposal that the TVM-HTX-diminished voiding resistance was accompanied by a decreased pressure required for driving urine flow, and thereby lessened the thermodynamic work of voiding.

### 4.4. TVM-HTX Increases Voiding Efficacy

Though the voiding pressure was decreased after TVM-HTX, the urodynamic data demonstrated that the voiding flow was consistently and significantly increased, implying the bladder capably drove urine with an adequate, or a higher, flow rate. Further analyses of the voided volume and voiding time revealed that, without affecting the voided volume, TVM-HTX shortened the voiding time. As the mean voiding flow was computed by the voided volume over the voiding time, and the operation-induced flow increment was correlated with the voiding time decrement rather than the change in voided volume, these findings not only imply that TVM-HTX post-operatively increased the flow rate of urine emission, but also support our proposal that the bladder post-operatively exhibited enhanced voiding efficacy as it prompted urine emission by reducing the voiding time with an almost unmodified voided volume. Collectively, all these findings suggest that TVM-HTX diminished the voiding resistance, which, on one hand, reduced the pressure gradient requisite for driving urine flow, and thereby lessened the workload of the bladder; and, on the other hand, prompted urine emission, which brought about an increased voiding efficacy.

### 4.5. Strengths and Limitations

As a retrospective study utilizing existing data, this investigation has inherent limitations in internal and external validity. The single-center study design with a relatively small sample size may introduce bias and limit the generalizability of the findings.

While mesh repair offers an efficacious anatomical reconstruction and satisfactory therapeutic outcome [35], it is also associated with potential side effects [36,37], particularly, mesh erosion [38,39]. As a long-term analysis has reported a 3.7% risk of erosions at 7 years after pelvic mesh repair [40], a long-term follow-up is warranted to confirm the durability of the benefits observed in this study, as our measurements were taken at an average of 135.20 ± 19.06 days after the procedure. Moreover, by comparing the effects of a conservative regimen and mid-urethra sling implantation on bladder functions, a very recent study demonstrates that the therapeutic benefit of the non-invasive option involves the activation of a hypogastric tone [41]; therefore, the potential role of a neural mechanism in the effects observed in this study needs future studies. When compared with the Rvod of UP patients with grade III or IV in this study, a study shows that patients with symptomatic POP ≥ stage II manifest with a higher pre-operative Rvod [28]. Because that study did not stratify patients according to the type of prolapse, whether patients with specific types or advanced grades of POP are characterized by a higher Rvod deficit than UP needs further study. Moreover, after mesh repair, these patients display a Rvod value similar to the level measured in our study. Considering a hysterectomy was performed only when indicated in that study, whether the hysterectomy impacts trivially on the outcome of Rvod caused by mesh repair is a question that warrants future investigations.

## 5. Conclusions

In conclusion, our results reveal that TVM-HTX diminished voiding resistance, and, therefore, prompted urine emission to increase the voiding efficacy and reduce the pressure gradient requisite for driving urine flow, thereby lessening the voiding workload of high-grade UP patients. Our findings could be informative for clinicians when deciding on pelvic reconstruction for UP patients.

## Figures and Tables

**Table 1:** Characteristics and urodynamic data of patients.

	PRE	POST
**UD, days**	32.86 ± 9.64 (before)	135.20 ± 19.06 (after, NA)
**Rvod, cmH_2_O-s/mL**	4.47 ± 1.02 (1.00 ± 0.00)	2.68 ± 0.56 (0.68 ± 0.07 **)
**Pvod, cmH_2_O)**	25.79 ± 3.32 (1.00 ± 0.00)	22.68 ± 2.99 (0.91 ± 0.06 *)
**Fvod, mL/s**	7.52 ± 0.86 (1.00 ± 0.00)	10.58 ± 1.38 (1.55 ± 0.19 **)
**Vvod, mL**	418.61 ± 36.97 (1.00 ± 0.00)	433.04 ± 34.14 (1.08 ± 0.08)
**Tvod, s**	63.14 ± 7.20 (1.00 ± 0.00)	45.30 ± 3.85 (0.81 ± 0.10 **)
**Apv, cmH_2_O-mL**	9436.38 ± 1432.22 (1.00 ± 0.00)	8315.24 ± 1092.44 (0.92 ± 0.07 **)

Data are presented as mean ± SEM (% of control). PRE: pre-operation; POST: post-operation; UD: urodynamic investigation; Rvod: mean voiding resistance; Pvod: mean voiding pressure; Fvod: mean voiding flow; Vvod; voided volume; Tvod: voiding time; Apv: trajectory-enclosed area. NA: not applicable; * *p* < 0.05, ** *p* < 0.01 vs. PRE, all N = 15.

## Data Availability

The data that support the findings of this study are available from the corresponding author, upon reasonable request.

## Abbreviations

TVM-HTX: transvaginal mesh with total hysterectomy; UP: uterine prolapse; Rvod: voiding resistance; Pvod: voiding pressure; Fvod: voiding flow; Vvod: voided volume; Tvod: voiding time; Apv: trajectory-enclosed area of the pressure–volume loop; ΔRvod: Rvod change; ΔPvod: Pvod change; ΔFvod: Fvod change; ΔTvod: Tvod change; ΔVvod: Vvod change; ΔApv: Apv change; POP: pelvic organ prolapse; PVA: pressure–volume analysis.
